# Evaluation of four novel genetic variants affecting hemoglobin A1c levels in a population-based type 2 diabetes cohort (the HUNT2 study)

**DOI:** 10.1186/1471-2350-12-20

**Published:** 2011-02-04

**Authors:** Jens K Hertel, Stefan Johansson, Helge Ræder, Carl GP Platou, Kristian Midthjell, Kristian Hveem, Anders Molven, Pål R Njølstad

**Affiliations:** 1 Department of Clinical Medicine, University of Bergen, Bergen, Norway; 2 Center for Medical Genetics and Molecular Medicine, Haukeland University Hospital, Bergen, Norway; 3 Department of Pediatrics, Haukeland University Hospital, Bergen, Norway; 4 HUNT Research Centre, Department of Public Health and General Practice, Norwegian University of Science and Technology, Levanger, Norway; 5 Department of Internal Medicine, Levanger Hospital, Nord-Trøndelag Health Trust, Levanger, Norway; 6 The Gade Institute, University of Bergen, Bergen, Norway; 7 Department of Pathology, Haukeland University Hospital, Bergen, Norway

## Abstract

**Background:**

Chronic hyperglycemia confers increased risk for long-term diabetes-associated complications and repeated hemoglobin A1c (HbA1c) measures are a widely used marker for glycemic control in diabetes treatment and follow-up. A recent genome-wide association study revealed four genetic loci, which were associated with HbA1c levels in adults with type 1 diabetes. We aimed to evaluate the effect of these loci on glycemic control in type 2 diabetes.

**Methods:**

We genotyped 1,486 subjects with type 2 diabetes from a Norwegian population-based cohort (HUNT2) for single-nucleotide polymorphisms (SNPs) located near the *BNC2*, *SORCS1*, *GSC *and *WDR72 *loci. Through regression models, we examined their effects on HbA1c and non-fasting glucose levels individually and in a combined genetic score model.

**Results:**

No significant associations with HbA1c or glucose levels were found for the *SORCS1*, *BNC2*, *GSC *or *WDR72 *variants (all *P*-values > 0.05). Although the observed effects were non-significant and of much smaller magnitude than previously reported in type 1 diabetes, the *SORCS1 *risk variant showed a direction consistent with increased HbA1c and glucose levels, with an observed effect of 0.11% (*P *= 0.13) and 0.13 mmol/l (*P *= 0.43) increase per risk allele for HbA1c and glucose, respectively. In contrast, the *WDR72 *risk variant showed a borderline association with reduced HbA1c levels (*β *= -0.21, *P *= 0.06), and direction consistent with decreased glucose levels (*β *= -0.29, *P *= 0.29). The allele count model gave no evidence for a relationship between increasing number of risk alleles and increasing HbA1c levels (*β *= 0.04, *P *= 0.38).

**Conclusions:**

The four recently reported SNPs affecting glycemic control in type 1 diabetes had no apparent effect on HbA1c in type 2 diabetes individually or by using a combined genetic score model. However, for the *SORCS1 *SNP, our findings do not rule out a possible relationship with HbA1c levels. Hence, further studies in other populations are needed to elucidate whether these novel sequence variants, especially rs1358030 near the *SORCS1 *locus, affect glycemic control in type 2 diabetes.

## Background

Good glycemic control may slow or prevent long-term diabetes-associated complications, preserve β-cell function, and improve long-term outcomes in both type 1 and type 2 diabetes [1,2]. Chronic hyperglycemia is also a risk factor for cardiovascular disease and all-cause mortality in persons without diabetes [3,4]. Individuals with diabetes often have difficulties attaining the recommended HbA1c goals, and inter- and intra-individual variability in HbA1c is commonly observed, even for patients using the same treatment regimen. Medical conditions that influence erythrocyte turnover, as well as genetic hereditary anemia and iron storage disorders, affect the HbA1c level. Moreover, several twin and family studies have demonstrated a heritable component in both HbA1c and fasting blood glucose levels, but these measures are not genetically correlated to each other [5-7]. Although emerging data now suggest that also common genetic variants may affect HbA1c and fasting glucose in both diabetic and non-diabetic individuals via both glycemic and non-glycemic pathways [5,8-19], little is known about the genetic background of HbA1c in type 2 diabetes.

Recently, Paterson and colleagues conducted a genome-wide association study (GWAS) on longitudinal repeated measures of HbA1c in 1,441 patients with type 1 diabetes collected from the Diabetes Control and Complications Trial (DCCT). They reported evidence of one major locus for glycemic control near *SORCS1*, as measured by both HbA1c and glucose, and three other loci (near *BNC2*, *GSC *and *WDR72*) achieving association close to genome-wide significance [20]. The clinical and biological significance of these findings remains to be demonstrated. They may, however, point to new pathways relevant for glycemic physiology [21]. We aimed to evaluate the individual and cumulative effect of the four novel loci on glycemic control in unselected individuals with type 2 diabetes collected from a Norwegian population-based study (HUNT2).

## Methods

### HUNT2 subjects and ethics

The study population has recently been described [22-24]. In short, the participants were ≥20 years of age (range 21-97) and comprised the total diabetes population drawn from an extensive population-based study (the HUNT2 Study). Diagnosis of diabetes was self-reported or identified by standard tests if random glucose was >8.0 mmol/l. Genomic DNA was available for 1,850 (94%) diabetic participants. Eight subjects with genetically verified maturity-onset diabetes of the young [24] and 205 subjects evaluated as having type 1 diabetes were excluded. More detailed inclusion and exclusion criteria for the diabetic participants have been described previously [22]. Of the 1,637 type 2 diabetic participants enrolled in the study, 73 subjects had missing data on HbA1c and another 44 subjects had missing BMI data. For those subjects with data, the range was 4.1-16.7% and 16.9-49.5 kg/m^2 ^for HbA1c and BMI, respectively. In addition, 34 individuals were excluded due to low genotyping quality or missing DNA. The study group finally consisted of 1,486 individuals with type 2 diabetes. The study was approved by the Regional Committee for Research Ethics and the Norwegian Data Inspectorate, and was performed according to the latest version of the Helsinki Declaration. All participants gave written informed consent.

### SNP selection, genotyping and quality control

We included only the four SNPs from Paterson et al. [20] which had shown the strongest association with glycemic control in type 1 diabetes. These are the non-coding SNPs rs10810632, rs1358030, rs11624318 and rs566369 located in or close to the *BNC2*, *SORCS1*, *GSC *and *WDR72 *genes, respectively. The genotyping was carried out by the multiplex MassARRAY^® ^*iPLEX*™ System (SEQUENOM Inc., San Diego, CA, USA) at the technology platform CIGENE, Ås, Norway. The final genotyping success rate was >95% for each SNP, with an average of 98.5%. For the internal controls, the genotyping concordance rate was 100% (n = 80 concordant calls). All SNPs examined were consistent with Hardy-Weinberg equilibrium (*P *> 0.05).

### Statistical analysis

We assessed the effect of each risk variant on single cross-sectional HbA1c levels and on non-fasting glucose levels using linear regression models assuming additive effects of allele dosage. Subsequently, we studied the combined SNP effect by using an allele counting method to assign a genetic risk score to each subject according to the total number of risk alleles that they carried. The allele counting method assumed equal and additive effects for each of the different variants. All analyses were conducted using age, sex and BMI as covariates, and none of the phenotypes analyzed were logarithmically transformed since a transformation did not influence the distributions and results noticeably. Detailed information regarding medical treatment was not available. Since our results represents a basic replication of previously reported findings, *P*-values presented in this study are two-sided, but was not corrected for the number of test performed. All analyses were carried out using the PLINK software [25] and Stata SE v10.0 for Windows (Stata Corp LP, Brownsville, TX, USA). We had >80% power to detect a total QTL variance of ≥0.5% at the 0.05-level, assuming additive effects, allele frequency of 0.1 or more [26].

## Results

Table 1 shows the clinical characteristics for the 1,486 individuals analyzed in the present study. Age, sex and BMI were included in the regression models as covariates. The risk alleles were defined according to Paterson et al. [20] and we assumed an additive model for all four SNPs throughout this study, based on the results reported in the DCCT study [20]. The results did not change notably in view of dominant or recessive genetic models (not shown). We observed allele frequencies similar to the frequencies reported in individuals with type 1 diabetes [20]. The mean HbA1c by genotype for each of the SNPs are presented in Table 2.

**Table 1:** Clinical characteristics of the 1,486 type 2 diabetic participants included in the study.

Individuals (*n*)	1,486
Sex (male/female)	706/780
Age (years at examination)	68.1 ± 11.9
BMI (kg/m^2^)	29.2 ± 4.8
HbA1c (%)	8.1 ± 1.8
Non-fasting serum glucose (mmol/l)	9.6 ± 4.2
Serum triglyceride (mmol/l)	2.5 ± 1.6
Serum cholesterol (mmol/l)	6.2 ± 1.3
Serum HDL cholesterol (mmol/l)	1.2 ± 0.4

Values are presented as means ± SD

**Table 2:** Genotype-specific means for single cross-sectional HbA1c levels in 1,486 subjects with type 2 diabetes.

Nearest gene	SNP	Common homozygote			Heterozygote			Rare homozygote			Minor allele*	Major allele*	MAF	MISS #
						
		N	Mean	SD	N	Mean	SD	N	Mean	SD				
*BNC2*	rs10810632	1215	8.05	0.52	257	8.12	0.11	13	7.95	0.65	**C**	T	0.09	1
*SORCS1*	rs1358030	648	7.94	0.07	622	8.16	0.07	139	8.02	0.16	**C**	T	0.32	77
*GSC*	rs11624318	908	8.07	0.06	500	8.02	0.08	76	8.11	0.21	A	**C**	0.22	2
*WDR72*	rs566369	1218	8.02	0.05	251	8.18	0.12	10	8.89	0.74	A	**G**	0.09	7

Risk alleles are defined according to Paterson et al. [20], and underlined and highlighted in bold.

MAF = minor allele frequency

MISS# = number of individuals with missing genotype data

* Alleles are indexed from the forward strand of the human reference sequence NCBI Build 36.

In the individual SNP analysis, none of the risk alleles reached statistical significance with either increased HbA1c measures or increased non-fasting serum glucose levels (all *P*-values > 0.05, Table 3). Although the observed effects were non-significant and of much smaller magnitude than previously reported in type 1 diabetes, the *SORCS1 *risk variant showed a direction consistent with increased HbA1c and glucose levels, with an observed effect of 0.11% (*P *= 0.13) and 0.13 mmol/l (*P *= 0.43) increase per risk allele for HbA1c and glucose, respectively (Table 3). In contrast, the *WDR72 *risk variant showed a borderline association with reduced HbA1c levels (*β *= -0.21, *P *= 0.06, Table 3), and direction consistent with decreased glucose levels (*β *= -0.29, *P *= 0.29, Table 3).

**Table 3:** Effects observed for the individual risk alleles and for the combined genetic scores on HbA1c and non-fasting serum glucose levels in 1,486 individuals with type 2 diabetes.

Individual SNP effects			HbA1c				Non-fasting serum glucose			
**Gene region**	**SNP**	**RAF**	**Effect size**	**Std Error**	**P-value**	**Sample size**	**Effect size**	**Std Error**	**P-value**	**Sample size**

*BNC2*	rs10810632	0.09 (C)	0.07	0.11	0.57	1485	-0.00	0.26	0.99	1484
*SORCS1*	rs1358030	0.32 (C)	0.11	0.07	0.13	1409	0.13	0.17	0.43	1408
*GSC*	rs11624318	0.78 (C)	0.03	0.08	0.75	1484	-0.14	0.18	0.45	1483
*WDR72*	rs566369	0.91 (G)	-0.21	0.12	0.06	1479	-0.29	0.27	0.29	1478

**Combined SNP effect based upon an allele count score**			0.04	0.04	0.38	1403	-0.05	0.1	0.66	1402

All effect sizes represent the change in HbA1c or non-fasting serum glucose per risk allele. Age, sex and BMI were included as covariates in the regression models. *P *values are two-sided and are unadjusted for multiple testing.

RAF: risk allele frequency

**Figure 1:**
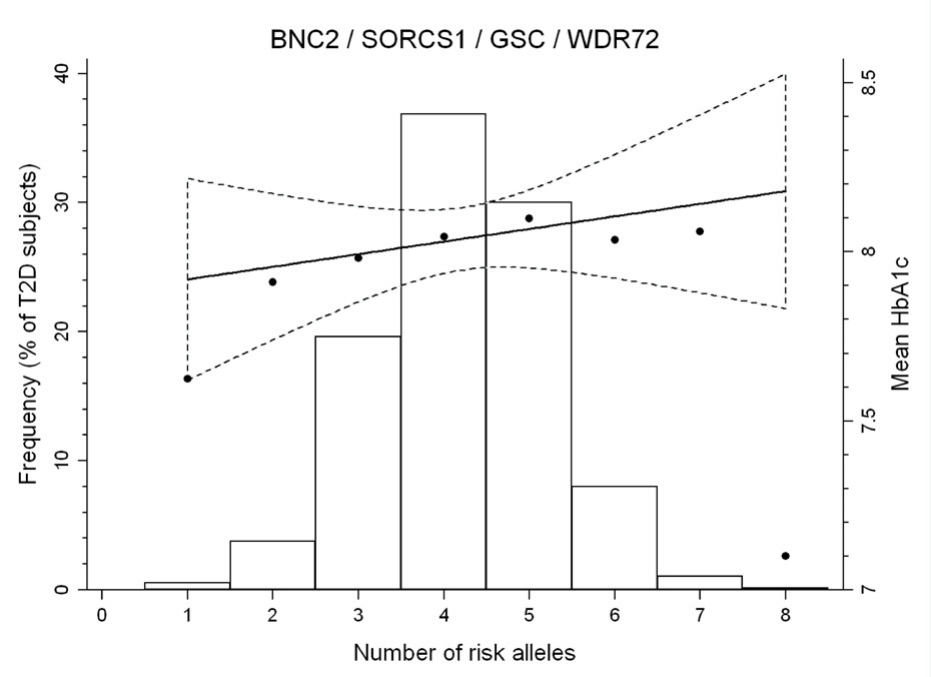
**Mean HbA1c (black circles) and frequency (bars) of type 2 diabetes individuals plotted against the number of risk alleles carried, and the relationship between *BNC2 *(rs10810632), *SORCS1 *(rs1358030), *GSC *(rs11624318) and *WDR72 *(rs566369) combined genotypes and mean HbA1c**. Only individuals genotyped for all variants are included (*n *= 1,403). The black line is the fitted HbA1c linear regression line with the area between the dashed curves representing the 95% confidence interval.

Even though the four examined loci were not significantly associated with increased HbA1c values at an individual level, three of the four risk variants showed concordance in allelic direction in which individuals carrying the risk allele had higher HbA1c. When we included all four variants in a combined genetic score model we observed, however, no evidence for a relationship between increasing number of risk alleles and increasing HbA1c levels (*P *= 0.38). Each additional risk allele demonstrated an increase in HbA1c of approximately 0.04% (Table 3, Figure 1).

## Discussion

To our knowledge, this study is the first attempt to evaluate the effect of the SNPs found by Paterson [20] with regard to glycemic control in type 2 diabetes. None of the SNPs were found associated with glycemic control in type 2 diabetes, either individually or combined by applying an allele count score. Hence, we were not able to confirm the strong associations recently reported in the DCCT genome-wide association study for HbA1c in the context of treated type 1 diabetes [20].

Using the same definition of the risk alleles as the DCCT study, the *WDR72 *SNP showed a borderline association with reduced and not increased HbA1c levels in our study. Thus, our results do not support a role of the *WDR72 *SNP on glycemic control as found in the DCCT study. The different pathophysiology between type 1 and type 2 diabetes could be one of the explanations why our results do not lend support to the finding that the four SNPs reported in the DCCT genome-wide association study [20] are genetic susceptibility factors for glycemic control in type 2 diabetes. In addition to a strong association with HbA1c, the *BNC2 *and *SORCS1 *risk alleles have revealed associations with mean glucose levels in type 1 diabetes [20], suggesting that these genetic variations affect HbA1c through their effects on glucose. We obtained no support for any associations between the *BNC2 *and *SORCS1 *SNPs and non-fasting serum glucose. The *SORCS1 *risk allele indicated, however, an effect consistent in direction with its effect on HbA1c.

There are some prior data supporting a role of the *SORCS1 *gene in glycemic traits. *SORCS1 *encodes a sortilin-related vacuolar protein sorting 10 domain-containing receptor, which binds to platelet-derived growth factor. A quantitative trait locus for fasting insulin in the syntenic region in mice has been described [27], with further independent evidence obtained in rats for post-intra-peritoneal glucose tolerance [28]. Two studies have also demonstrated modest evidence for association between SNPs in *SORCS1 *and fasting insulin, insulin sensitivity and insulin resistance in humans [29,30]. However, no association has been found with type 2 diabetes. Considering our results in light of the previous reported results and features for *SORCS1*, we can not refute a possible link between *SORCS1 *and glycemic control in type 2 diabetes.

The *BNC2*, *WDR72 *and *GSC *genes encode a zinc finger protein, a putative β propeller expected to be involved in protein-protein interactions and a transcription factor of the paired homeobox family of proteins, respectively. Their exact function is unknown. Except for the results reported by Paterson and colleagues [20] none of these gene regions have previously been shown to be associated with glycemic traits in humans or animals. We found no evidence of association for any of these loci with glycemia in our type 2 diabetes cohort. The possibility nevertheless exists that the analysed SNP or genetic variants in strong linkage disequilibrium with these SNPs, are involved in glycemia, but that they have weak effects and/or are population specific. Our results therefore emphasize the need for further replication studies if one is to be successful in defining the true genetic risk factors involved in glycemic-related traits.

There are limitations of our study. We had access to only one HbA1c and non-fasting blood glucose value for each case, in contrast to the repeated measurements used by the DCCT investigators during the course of a carefully controlled clinical trial. Furthermore, the use of HbA1c as a quantitative trait modulated by genetic factors must be taken with caution in the context of pharmacological treatment, since treatment as an environmental variable may overwhelm the genetic signal. Whereas the DCCT investigators attempted to control for this, we had no access to information on medical treatment in the current study. Thus, our data may be confounded by environmental factors and cannot be considered a straight-forward replication study.

Our study has, however, also several important strengths. The HUNT cohort is a well-characterized, stable (net emigration around 0.3% per year) and ethnically uniform (less than 3% of the people are of non-Caucasian origin) population from a clearly defined region of Norway [31]. Our study participants were part of an all-population-inclusive survey with high attendance. Hence, possible selection biases that can arise when studying referral patients or patients selected for inclusion in clinical intervention studies were avoided. The HUNT samples have previously been validated by genotyping of known type 2 diabetes risk variants [23,32] indicating that the HUNT population contains a representative diabetes cohort. Furthermore, we observed allele frequencies similar to the frequencies reported by Paterson et al [20], arguing against problems with population stratification. Finally, our study was conducted in one data set avoiding loss of power and, although the design was different than that of the initial report [20], we tested identical SNPs.

## Conclusions

The four recently reported loci affecting glycemic control in type 1 diabetes patients had no apparent effect on HbA1C levels in type 2 diabetes, neither individually nor by using a combined genetic score model. For the *SORCS1 *SNP however, we cannot refute a possible relationship with HbA1c. Hence, further studies in other populations are needed to elucidate whether these novel sequence variants, especially rs1358030 near the *SORCS1 *locus, affect glycemic control in type 2 diabetes.

## Abbreviations

DCCT: Diabetes Control and Complications Trial; GWA: genome-wide association; HbA1c: glycosylated hemoglobin; HUNT: Helseundersøkelsen i Nord-Trøndelag; SNP: single-nucleotide polymorphism.

## Competing interests

The authors declare that they have no competing interests.

## Authors' contributions

JKH contributed to the study design, performed the statistical analyses, researched and interpreted the data, and wrote the manuscript. SJ contributed to the study design, directed the genotyping analyses, researched and interpreted the data, involved in drafting the manuscript. HR assisted in the study design, researched data, contributed to discussion and reviewed and edited the manuscript. CGPP researched data, contributed to discussion, and reviewed and edited the manuscript. KM contributed to discussion, and reviewed and edited the manuscript. KH contributed to discussion, and reviewed and edited the manuscript. AM assisted with study design, interpreted the data, contributed to discussion and helped to draft the manuscript. PRN conceived of the study, participated in the study design and coordination, interpreted the data, contributed to discussion and helped to draft the manuscript. All authors read and approved the final manuscript.

## Pre-publication history

The pre-publication history for this paper can be accessed here:

http://www.biomedcentral.com/1471-2350/12/20/prepub

## References

[B1] Gaede P Lund-Andersen H Parving HH Pedersen O Effect of a multifactorial intervention on mortality in type 2 diabetes N Engl J Med 2008 358 6 580 591 10.1056/NEJMoa0706245 18256393

[B2] Reichard P Nilsson BY Rosenqvist U The effect of long-term intensified insulin treatment on the development of microvascular complications of diabetes mellitus N Engl J Med 1993 329 5 304 309 10.1056/NEJM199307293290502 8147960

[B3] Balkau B Shipley M Jarrett RJ Pyorala K Pyorala M Forhan A Eschwege E High blood glucose concentration is a risk factor for mortality in middle-aged nondiabetic men. 20-year follow-up in the Whitehall Study, the Paris Prospective Study, and the Helsinki Policemen Study Diabetes Care 1998 21 3 360 367 10.2337/diacare.21.3.360 9540016

[B4] Barr EL Boyko EJ Zimmet PZ Wolfe R Tonkin AM Shaw JE Continuous relationships between non-diabetic hyperglycaemia and both cardiovascular disease and all-cause mortality: the Australian Diabetes, Obesity, and Lifestyle (AusDiab) study Diabetologia 2009 52 3 415 424 10.1007/s00125-008-1246-y 19130039

[B5] Meigs JB Panhuysen CI Myers RH Wilson PW Cupples LA A genome-wide scan for loci linked to plasma levels of glucose and HbA(1c) in a community-based sample of Caucasian pedigrees: The Framingham Offspring Study Diabetes 2002 51 3 833 840 10.2337/diabetes.51.3.833 11872688

[B6] Simonis-Bik AM Eekhoff EM Diamant M Boomsma DI Heine RJ Dekker JM Willemsen G van Leeuwen M de Geus EJ The heritability of HbA1c and fasting blood glucose in different measurement settings Twin Res Hum Genet 2008 11 6 597 602 10.1375/twin.11.6.597 19016616

[B7] Snieder H Sawtell PA Ross L Walker J Spector TD Leslie RD HbA(1c) levels are genetically determined even in type 1 diabetes: evidence from healthy and diabetic twins Diabetes 2001 50 12 2858 2863 10.2337/diabetes.50.12.2858 11723071

[B8] An P Freedman BI Hanis CL Chen YD Weder AB Schork NJ Boerwinkle E Province MA Hsiung CA Wu X Genome-wide linkage scans for fasting glucose, insulin, and insulin resistance in the National Heart, Lung, and Blood Institute Family Blood Pressure Program: evidence of linkages to chromosome 7q36 and 19q13 from meta-analysis Diabetes 2005 54 3 909 914 10.2337/diabetes.54.3.909 15734873

[B9] Bonnefond A Vaxillaire M Labrune Y Lecoeur C Chevre JC Bouatia-Naji N Cauchi S Balkau B Marre M Tichet J Genetic variant in HK1 is associated with a proanemic state and A1C but not other glycemic control-related traits Diabetes 2009 58 11 2687 2697 10.2337/db09-0652 19651813 PMC2768183

[B10] Bouatia-Naji N Bonnefond A Cavalcanti-Proenca C Sparso T Holmkvist J Marchand M Delplanque J Lobbens S Rocheleau G Durand E A variant near MTNR1B is associated with increased fasting plasma glucose levels and type 2 diabetes risk Nat Genet 2009 41 1 89 94 10.1038/ng.277 19060909

[B11] Bouatia-Naji N Rocheleau G Van Lommel L Lemaire K Schuit F Cavalcanti-Proenca C Marchand M Hartikainen AL Sovio U De Graeve F A polymorphism within the G6PC2 gene is associated with fasting plasma glucose levels Science 2008 320 5879 1085 1088 10.1126/science.1156849 18451265

[B12] Chen WM Erdos MR Jackson AU Saxena R Sanna S Silver KD Timpson NJ Hansen T Orru M Grazia Piras M Variations in the G6PC2/ABCB11 genomic region are associated with fasting glucose levels J Clin Invest 2008 118 7 2620 2628 18521185 10.1172/JCI34566 PMC2398737

[B13] Meigs JB Manning AK Fox CS Florez JC Liu C Cupples LA Dupuis J Genome-wide association with diabetes-related traits in the Framingham Heart Study BMC Med Genet 2007 8 Suppl 1 S16 10.1186/1471-2350-8-S1-S16 17903298 PMC1995610

[B14] Pare G Chasman DI Parker AN Nathan DM Miletich JP Zee RY Ridker PM Novel association of HK1 with glycated hemoglobin in a non-diabetic population: a genome-wide evaluation of 14,618 participants in the Women's Genome Health Study PLoS Genet 2008 4 12 e1000312 10.1371/journal.pgen.1000312 19096518 PMC2596965

[B15] Prokopenko I Langenberg C Florez JC Saxena R Soranzo N Thorleifsson G Loos RJ Manning AK Jackson AU Aulchenko Y Variants in MTNR1B influence fasting glucose levels Nat Genet 2009 41 1 77 81 10.1038/ng.290 19060907 PMC2682768

[B16] Sabatti C Service SK Hartikainen AL Pouta A Ripatti S Brodsky J Jones CG Zaitlen NA Varilo T Kaakinen M Genome-wide association analysis of metabolic traits in a birth cohort from a founder population Nat Genet 2009 41 1 35 46 10.1038/ng.271 19060910 PMC2687077

[B17] Dupuis J Langenberg C Prokopenko I Saxena R Soranzo N Jackson AU Wheeler E Glazer NL Bouatia-Naji N Gloyn AL New genetic loci implicated in fasting glucose homeostasis and their impact on type 2 diabetes risk Nat Genet 2010 42 2 105 116 10.1038/ng.520 20081858 PMC3018764

[B18] Orho-Melander M Melander O Guiducci C Perez-Martinez P Corella D Roos C Tewhey R Rieder MJ Hall J Abecasis G Common missense variant in the glucokinase regulatory protein gene is associated with increased plasma triglyceride and C-reactive protein but lower fasting glucose concentrations Diabetes 2008 57 11 3112 3121 10.2337/db08-0516 18678614 PMC2570409

[B19] Soranzo N Sanna S Wheeler E Gieger C Radke D Dupuis J Bouatia-Naji N Langenberg C Prokopenko I Stolerman E Common variants at ten genomic loci influence hemoglobin A1C levels via glycemic and non-glycemic pathways Diabetes 2010 59 12 3229 3239 10.2337/db10-0502 20858683 PMC2992787

[B20] Paterson AD Waggott D Boright AP Hosseini SM Shen E Sylvestre MP Wong I Bharaj B Cleary PA Lachin JM A genome-wide association study identifies a novel major locus for glycemic control in type 1 diabetes, as measured by both A1C and glucose Diabetes 2010 59 2 539 549 10.2337/db09-0653 19875614 PMC2809960

[B21] Florez JC A Genome-Wide Association Study of Treated A1C - A Genetic Needle in an Environmental Haystack? Diabetes 2010 59 2 332 334 10.2337/db09-1636 20103712 PMC2809959

[B22] Johansson S Raeder H Eide SA Midthjell K Hveem K Sovik O Molven A Njolstad PR Studies in 3,523 Norwegians and meta-analysis in 11,571 subjects indicate that variants in the hepatocyte nuclear factor 4 alpha (HNF4A) P2 region are associated with type 2 diabetes in Scandinavians Diabetes 2007 56 12 3112 3117 10.2337/db07-0513 17827402

[B23] Hertel JK Johansson S Raeder H Midthjell K Lyssenko V Groop L Molven A Njolstad PR Genetic analysis of recently identified type 2 diabetes loci in 1,638 unselected patients with type 2 diabetes and 1,858 control participants from a Norwegian population-based cohort (the HUNT study) Diabetologia 2008 51 6 971 977 10.1007/s00125-008-0982-3 18437351

[B24] Eide SA Raeder H Johansson S Midthjell K Sovik O Njolstad PR Molven A Prevalence of HNF1A (MODY3) mutations in a Norwegian population (the HUNT2 Study) Diabet Med 2008 25 7 775 781 10.1111/j.1464-5491.2008.02459.x 18513305

[B25] Purcell S Neale B Todd-Brown K Thomas L Ferreira MA Bender D Maller J Sklar P de Bakker PI Daly MJ PLINK: a tool set for whole-genome association and population-based linkage analyses Am J Hum Genet 2007 81 3 559 575 10.1086/519795 17701901 PMC1950838

[B26] Purcell S Cherny SS Sham PC Genetic Power Calculator: design of linkage and association genetic mapping studies of complex traits Bioinformatics 2003 19 1 149 150 10.1093/bioinformatics/19.1.149 12499305

[B27] Clee SM Yandell BS Schueler KM Rabaglia ME Richards OC Raines SM Kabara EA Klass DM Mui ET Stapleton DS Positional cloning of Sorcs1, a type 2 diabetes quantitative trait locus Nat Genet 2006 38 6 688 693 10.1038/ng1796 16682971

[B28] Granhall C Park HB Fakhrai-Rad H Luthman H High-resolution quantitative trait locus analysis reveals multiple diabetes susceptibility loci mapped to intervals < 800 kb in the species-conserved Niddm1i of the GK rat Genetics 2006 174 3 1565 1572 10.1534/genetics.106.062208 16951059 PMC1667097

[B29] Florez JC Manning AK Dupuis J McAteer J Irenze K Gianniny L Mirel DB Fox CS Cupples LA Meigs JB A 100K genome-wide association scan for diabetes and related traits in the Framingham Heart Study: replication and integration with other genome-wide datasets Diabetes 2007 56 12 3063 3074 10.2337/db07-0451 17848626

[B30] Goodarzi MO Lehman DM Taylor KD Guo X Cui J Quinones MJ Clee SM Yandell BS Blangero J Hsueh WA SORCS1: a novel human type 2 diabetes susceptibility gene suggested by the mouse Diabetes 2007 56 7 1922 1929 10.2337/db06-1677 17426289

[B31] Holmen J Midthjell K Krüger Ø Langhammer A Holmen T Bratberg G Vatten L Lund-Larsen P The Nord-Trøndelag Health Study 1995-97 (HUNT2): Objectives, contents, methods and participation Norw J Epidemiol 2003 13 19 32

[B32] Thorsby PM Midthjell K Gjerlaugsen N Holmen J Hanssen KF Birkeland KI Berg JP Comparison of genetic risk in three candidate genes (TCF7L2, PPARG, KCNJ11) with traditional risk factors for type 2 diabetes in a population-based study--the HUNT study Scand J Clin Lab Invest 2009 69 2 282 287 10.1080/00365510802538188 18972257

